# Supplementing rumen‐protected methionine to lactating multiparous dairy cows did not improve reproductive performance

**DOI:** 10.1111/rda.13509

**Published:** 2019-08-06

**Authors:** David Süss, Michael Iwersen, Vanessa Schweinzer, Erika Gusterer, Peter Kanz, Stefanie Krieger, Harald Pothmann, Karen Wagener, Michael Hoelker, Dawit Tesfaye, Karl Schellander, Ariane Helmbrecht, Claudia Parys, Marc Drillich

**Affiliations:** ^1^ Clinical Unit for Herd Health Management in Ruminants, Department for Farm Animals and Veterinary Public Health, University Clinic for Ruminants University of Veterinary Medicine Vienna Vienna Austria; ^2^ Research Station Frankenforst, Faculty of Agriculture University of Bonn Königswinter Germany; ^3^ Institute of Animal Science, Animal breeding and Husbandry University of Bonn Bonn Germany; ^4^ Evonik Nutrition & Care GmbH Hanau Germany

**Keywords:** amino acid, dairy cattle, fertility, methionine, reproduction

## Abstract

There is evidence that supplementing methionine has positive effects on uterine environment, oocyte quality and embryo development in cattle. Thus, the objective of this study was to evaluate reproductive traits of cows supplemented with rumen‐protected methionine (RPM) during early to mid‐lactation in comparison with an untreated control group (CON). An additional focus was on the effect of puerperal diseases on reproductive performance parameters in RPM‐supplemented group MET and in CON. A total of 1,709 multiparous Holstein‐Friesian cows were enrolled in this field trial conducted on a commercial dairy farm in Slovakia. Cows were allocated at approximately 12 days post‐partum (dpp) to either CON or MET, the latter supplemented with 25.0 g–27.2 g RPM per cow per day incorporated into the total mixed ration (TMR) until leaving the study pen at approximately 140 dpp. The amount of RPM was calculated based on individual feed ingredients analysis and adjusted during the study period when TMR changed. Cows were monitored during the post‐partum period by vaginal examination (day 5 pp), measuring of beta‐hydroxybutyrate in blood (3, 5, and 8 dpp) and by vaginal examination, uterine cytology and measuring of back fat thickness by ultrasound (all at 31 ± 3 dpp).

Compared with CON, cows supplemented with RPM did not show better reproduction performance parameters (first service submission rate, days to first service, conception risk, days open 140). Results from binary logistic regression model for the risk of conception showed that metritis had a significant effect, but the supplementation of methionine had not. Results of Cox regression analysis for the odds of conception within 140 dpp revealed only metritis and clinical endometritis as significant factors. In conclusion, supplementation of RPM had no beneficial effect on reproductive performance in this study farm compared with an untreated control group.

## 1 INTRODUCTION

The intensive genetic selection for high milk yields in the last decades has reduced fertility, due to poor expression of oestrus, uterine infections, defective embryos and oocytes and other post‐partum clinical problems (Dobson, Smith, Royal, Knight, & Sheldon, 2007). Important nutritional elements in dairy cow reproduction are amino acids (AA). Several studies have postulated a central role of AA for the development of the bovine conceptus (Groebner et al., 2011; Hugentobler et al., 2007). In lactating dairy cows, methionine is the most limiting AA (National Research Council, 2001). To prevent its degradation by microorganisms in the rumen, it needs to be supplemented as rumen‐protected methionine (RPM). It has been demonstrated that supplementing RPM leads to a greater lipid accumulation in the preimplantation embryo, which serves as an energy substrate and enhances the embryo's capacity for survival, when RPM was supplemented between 21 days before calving to 30 days post‐partum (Acosta et al., 2016). Moreover, Acosta et al. (2017) found higher concentrations of methionine in the follicular fluid of the first dominant follicle post‐partum in cows supplemented with RPM and rumen‐protected choline between 21 days before calving to 30 days post‐partum and assumed that higher methionine concentrations in the follicular fluid could affect oocyte quality. Furthermore, there is evidence that supplementing RPM from 21 days prepartum to 73 days post‐partum improves uterine immune function by positive effects on neutrophil infiltration, glandular morphology and neutrophil extracellular trap formation (Stella et al., 2018). Studies that tested effects of RPM on fertility on a herd level, however, are rare. Recently, Toledo et al. (2017) found that daily top‐dressing of 21.2 g RPM from 30 to 126 days post‐partum can improve embryo development and reduce pregnancy losses in multiparous cows, but did not affect pregnancy per artificial insemination. Although several authors (Acosta et al., 2016, 2017; Stella et al., 2018) postulated positive effects of RPM on fertility supplemented already at the beginning of the transition period, also some publications (Peñagaricano et al., 2013; Toledo et al., 2017) stated positive effects of RPM fed only post‐partum. Therefore, the beginning of the supplementation seems also decisive. Thus, there is a need to investigate the effects of methionine supplementation on reproductive performance on herd level. The hypothesis of our study was that supplementation of 25.0 to 27.2 g RPM incorporated into the total mixed ration during early to mid‐lactation improves reproductive performance in dairy cows at the herd level in comparison with an untreated control group. Furthermore, we analysed the effect of puerperal diseases on reproductive performance on both groups.

## 2 MATERIALS AND METHODS

### 2.1 Experimental design

The project was approved by the Slovakian Regional Veterinary Food Administration, as well as by the institutional ethics committee of the University of Veterinary Medicine Vienna, Austria (ETK‐09/02/2016).

This field trial was conducted from March 2016 to November 2017 on a commercial dairy farm in Slovakia, housing approximately 2,400 Holstein‐Friesian cows. Cows were milked twice daily in a rotary milking parlour. The average annual energy corrected milk yield was 9,260 kg (based on 4.0% butterfat and 3.4% protein). Only multiparous cows were enrolled in this study, because first lactation cows were kept on another farm site. After leaving the fresh cow group approximately 12 dpp (8–40 dpp), cows were kept in one free stall with a pen for control cows (CON) or another for methionine supplemented cows (MET). Pens were equipped with cubicles and concrete floors in groups of approximately 250 cows in each pen. For a randomized allocation, prior to parturition, matched pairs were built based on expected calving date, parity and previous lactation milk yield. Cows of the matched pairs were then randomly assigned to CON or MET using the “rand function” from Excel Version 14 (Microsoft Corp.).

In the middle of the study period, pens for CON and MET groups were switched in order to address possible environmental factors. Study animals in CON or MET that were moved to any other group, for example mastitis pen, sick pen or a wrong pen, and were not returned within 5 days to their originally assigned group were only used for statistical analyses until the last day before leaving the study pen.

Initially, 1,709 cows entered the study groups; however, 136 cows had to be excluded because they were classified as “do not breed”‐cows within 70 dpp and 11 cows because they were inseminated within the voluntary waiting period for artificial insemination (VWPAI). The number of cows eligible for the analysed parameters differed depending on the definition of the parameters and on the number of cows that were excluded because they left the groups for more than 5 days.

### 2.2 Feeding

All animals enrolled in the study received identical diets before they entered either the MET or CON pen, where MET ration was supplemented with RPM (Mepron^®^, Evonik Nutrition & Care GmbH) whereas cows in the CON pen received no RPM. The composition of the basal diet, mainly based on corn silage, alfalfa silage, wet distillers grains with solubles, corn gluten meal, corn‐cob mix, rapeseed extraction meal and beet pulp silage, was adjusted during the study period based on regular analyses of the total mixed ration (TMR). The ingredient compositions of the ration at the beginning and at the end of the study are shown in Table S1. At the beginning of the study, a Lys‐to‐Met ratio of 3.4:1 was determined in the basal diet using the AminoCow Dairy Ration Evaluater, version 3.5.2 (Evonik Industries; http://www.makemilknotmanure.com/aminocow.php). The target of methionine supply was a Lys‐to‐Met ratio close to 2.8:1 (Batistel et al., 2017; Zhou et al., 2016). Therefore, the calculated amount of RPM fed per cow ranged between 25.0 and 27.2 g RPM and was mixed with the mineral and vitamin premix and fed with the TMR twice a day in MET. For animals in CON, the amount of RPM in the mineral and vitamin premix was compensated by an increased quantity of carrier substances. Analyses of the mineral and vitamin premix revealed a concentration of 22.0 ± 3.7% RPM per kg premix. Depending on used quantities of the mineral and vitamin premix, the supplemented quantity of RPM was 26.8 ± 4.3 g per cow per day considering the entire study period. Mepron^®^ has an ethyl‐cellulose film coating and contains 85% DL‐methionine. The rumen bypass and intestinal digestibility coefficient of Mepron^®^ is 80% (Overton, LaCount, Cicela, & Clark, 1996) and 90% (Schwab, 1995), respectively. Therefore, study cows in MET received 6.1 g of methionine available for absorption per 10 g of Mepron^®^.

TMR was offered twice per day and adjusted daily to achieve refusals of 5–10%. Dry matter intake (DMI) was recorded daily for CON and MET with a near‐infrared spectroscopy system (Dinamica Generale) used by TMR mixer (Siloking, SelfLine System 1,000+ 3,023, Mayer Maschinenbau GmbH) and was 21.6 ± 1.5 kg for CON and 21.7 ± 1.5 kg for MET over the entire study period. Further details are presented in Table S2.

### 2.3 Reproductive management and reproductive performance parameters

The VWPAI was set at 50 dpp. All cows not bred by 70 dpp were subjected to an Ovsynch protocol (GnRH‐7 d‐PGF_2α_‐56 hrs‐GnRH‐16 to 18 hrs‐timed AI). Pregnancy was tested and confirmed by ultrasonography or by transrectal palpation of the uterus and its contents at 42 d ± 3 d, 93 d ± 3 d after AI and at drying off by farm veterinarians.

Analysed reproductive performance parameters included days to first service (DFS; number of days from calving to first AI), first service submission rate (FSSR; percentage of cows receiving at least one insemination in the first 3 weeks after VWPAI), first service conception risk (FSCR; number of first services resulting in pregnancies by total number of first services × 100), second service conception risk (SSCR; number of second services resulting in pregnancy by total number of second services × 100), total conception risk (TCR; number of all services resulting in pregnancies by total number of all services × 100), days open for cows pregnant within 140 dpp, that is before leaving study pens (DOPN140; days form calving to conception) and pregnancy losses after first pregnancy check.

### 2.4 Blood sampling, uterine health status and body condition

Health data in the early post‐partum period were collected to test effects on reproductive performance that might bias the effect of RPM. Blood samples were taken from each cow from a coccygeal vessel at 3, 5 and 8 dpp with vacuum tubes coated with a clot activator for serum collection (Vacuette, 9 ml, Greiner Bio‐One GmbH) and analysed with an electronic hand‐held device (FreeStyle Precision, Abbott GmbH & Co. KG) for ß‐hydroxybutyrate (BHB) concentration.

Vaginal discharge was evaluated at 5 dpp, and cows were classified as healthy or affected by metritis (>39.5°C puerperal metritis; ≤39.5°C clinical metritis) (Sheldon, Lewis, LeBlanc, & Gilbert, 2006). All cows were re‐checked at 31 ± 3 dpp by vaginal examination with a disposable rectal examination glove. A modified vaginal discharge score (VDS) (Williams et al., 2005) was used to classify vaginal mucus as (E0) clear mucus, (E1) ≤50% off‐white or white mucopurulent material and (E2) ≥50% off‐white or white mucopurulent material. Furthermore, uterine cytology samples were taken using the cytobrush method (Kasimanickam et al., 2004). Cytological samples were prepared by rolling the brush onto a clean glass microscope slide, fixed and stained (LT‐SYS, Labor und Technik) and evaluated under a microscope (× 400 magnification) by counting a total of 300 cells to determine the proportion of polymorphonuclear neutrophils (PMN) (Melcher, Prunner, & Drillich, 2014). A cut‐off value for the diagnosis of subclinical endometritis (SE) was set at 5% PMN (Madoz et al., 2013). Cows were classified as healthy (VDS = E0, PMN <5%) or affected by SE (VDS = E0, PMN ≥5%) or clinical endometritis (CE; VDS ≥E1).

Body condition was determined as back fat thickness (BFT) measured by ultrasound (Schröder & Staufenbiel, 2006) at 31 ± 3 dpp. For the analyses, cows were categorized into <14 mm and ≥14 mm BFT.

### 2.5 Statistical analysis

Statistical analyses were performed with Excel 2010 (Microsoft Corp.) and SPSS software (version 24.0, IBM SPSS Inc.). DFS and DOPN140 were compared between CON and MET by Mann–Whitney U test. For DOPN140 a Cox regression model and Kaplan–Meier survival analyses, censored for not pregnant cows or removed from the study, were calculated. Proportions were compared by chi‐square analysis. FSCR and TCR were evaluated with a binary logistic regression model, including group (0: CON; 1: MET), parity (0: 2nd lactation; 1: ≥ 3rd lactation), BHB‐level at 3, 5 and 8 dpp (0: normoketotic; 1: at least one day hyperketotic, ≥1.2 mmol/L BHB), uterine health status at 5 dpp (0: healthy; 1: metritis), SE (0: no; 1: yes), CE (0: no; 1: yes), BFT class (0:≥14 mm; 1:<14 mm) as factors. The level of significance was set at *α* = .05 for all statistical analyses.

## 3 RESULTS

### 3.1 Descriptive information about groups, health and milk yield

In CON and MET, 51.3% and 47.3% were in the 2nd lactation and 48.1 and 52.7% had ≥3rd lactations, respectively. Cows entered the study pens with a mean of 11.6 ± 5.1 dpp in CON and 12.0 ± 5.1 dpp in MET, and left the study pens at 139.1 ± 14.8 (CON) and 137.5 ± 17.2 dpp (MET). Results of the uterine health checks at 5 dpp and at 31 ± 3 dpp are presented in Table 1. No significant differences were found between groups, indicating a similar distribution of diseases prior to the start of feeding RPM, except the greater prevalence of SE in CON (14.6%) compared with MET (19.2%; *p* = .03). The proportion of hyperketotic cows in the first week post‐partum was 11.7% and 12.3% in CON and MET, respectively. The proportion of cows with <14 mm BFT was 81.9% and 82.6% in CON and MET, respectively.

**Table 1:** Descriptive statistics of the uterine health status at 5 dpp and 31 ± 3 dpp in CON and MET

Status	CON†		MET†		*P*‐value*
	*n*	*n* (%)	*n*	*n* (%)	
Healthy at 5 dpp	494	77.3	494	79.7	> .05
Clinical metritis	107	16.7	85	13.7	> .05
Puerperal metritis	23	3.6	26	4.2	> .05
Missing	15	2.3	15	2.4	> .05
Healthy at 31 ± 3 dpp	345	54.0	321	51.8	> .05
Subclinical endometritis	93	14.6	119	19.2	.03
Clinical endometritis	165	25.8	151	24.3	> .05
E1‡	109	17.0	92	14.8	> .05
E2‡	56	8.8	59	9.5	> .05
Missing	36	5.6	29	4.7	> .05

† CON = Control group, MET = Rumen‐protected methionine group.

‡ E1 ≤50% off‐white or white mucopurulent material, E2 ≥50% off‐white or white mucopurulent material.

* *P*‐value for comparison between CON and MET (*p* < .05).

Monthly milk test results during the study period (first three test data in the study group) revealed for CON 41.8 kg milk per day (3.09 protein %, 3.59 fat %) and for MET 41.5 kg milk per day (3.10 protein %, 3.52 fat %).

### 3.2 First service submission rate and days to first service

The analyses of FSSR comprised of 579 cows in CON and 572 in MET and revealed no differences between CON (41.8%) and MET (41.4%; *p* > .05). In CON, healthy animals (48.7%) and cows with SE (42.0%) had a significantly greater FSSR compared to cows with CE (26.2%). Cows with E1 (32.3%) had a significantly greater FSSR than with E2 (14.3%). MET cows with SE showed a significantly greater FSSR (53.2%) compared to healthy animals (42.5%) and cows with CE (31.5%), but healthy cows showed also a significantly improved FSSR compared to study animals with CE (Table 2).

**Table 2:** Reproductive performance parameters for cows with different uterine health status in CON and MET

Status	CON†		MET†		*P*‐value*
	*n*	*n* (%)	*n*	*n* (%)	
First service submission rate					
Overall	242/579	41.8	237/572	41.4	0.90
Healthy	153/314	48.7^a^	124/292	42.5^a^	0.12
Subclinical endometritis	37/88	42.0^c^	59/111	53.2^b,c^	0.12
Clinical endometritis	38/145	26.2^b,d^	45/143	31.5^b,d^	0.32
E1‡	31/96	32.3^e^	31/87	35.6	0.63
E2‡	7/49	14.3^f^	14/56	25.0	0.17
Missing	14/32	43.8	9/26	34.6	
First service conception risk					
Overall	209/470	44.5	184/446	41.3	0.33
Healthy	128/263	48.7^a^	95/227	41.9	0.13
Subclinical endometritis	33/74	44.6	41/91	45.1	0.95
Clinical endometritis	37/108	34.3^b^	39/110	35.5	0.85
E1	24/70	34.3	29/68	42.6^a^	0.31
E2	13/38	34.2	10/42	23.8^b^	0.31
Missing	11/25	44.0	9/18	50.0	
Second service conception risk					
Overall	54/123	43.9	52/113	46.0	0.74
Healthy	31/66	47.0	26/56	46.4	0.95
Subclinical endometritis	11/27	40.7	13/23	56.5	0.27
Clinical endometritis	9/25	36.0	9/25	36.0	1.00
E1	8/19	42.1	7/17	41.2	0.96
E2	1/6	16.7	2/8	25.0	0.71
Missing	3/5	60.0	4/9	44.4	
Total conception risk					
Overall	270/608	44.4	243/577	42.1	0.43
Healthy	164/340	48.2^a^	126/291	43.3	0.22
Subclinical endometritis	45/103	43.7	55/117	47.0^a^	0.62
Clinical endometritis	47/134	35.1^b^	49/142	34.5^b^	0.92
E1	33/90	36.7	37/90	41.1^c^	0.54
E2	14/44	31.8	12/52	23.1^d^	0.34
Missing	14/31	45.2	13/27	48.1	

Note

Values in columns with different superscripts (a,b;c,d;e,f) differ (*p* < .05).

† CON = Control group, MET = Rumen‐protected methionine group.

‡ E1 ≤50% off‐white or white mucopurulent material, E2 ≥50% off‐white or white mucopurulent material.

* *P*‐value for comparison between CON and MET (*p* < .05).

No significant difference was found for DFS between CON (73.0 ± 12.4) and MET (72.9 ± 12.4). This comprised 566 cows in CON and 551 cows in MET.

### 3.3 First service conception risk

No significant difference in FSCR was found between CON (44.5%, *n* = 470) and MET (41.3%, *n* = 446). In CON, significantly more healthy cows (48.7%) became pregnant after the first AI compared to cows with CE (43.3%). In MET, cows with E1 (42.6%) showed a significantly greater FSCR compared to cows with E2 (23.8%). Table 2 shows further details. Results of binary logistic regression analyses revealed metritis (hazard ration HR = 0.66; CI_95_ = 0.45–0.95; *p* = .03) as significant factor for non‐pregnancy after first service (Table 3).

**Table 3:** Results of binary logistic regression analyses for the risk of conception after first AI (*n* = 825) and more AI (*n* = 1,064)

Factors§	FSCR†			TCR‡		
	Hazard ratio	95% CI	*P*‐value*	Hazard ratio	95% CI	P‐value*
Study group	0.86	0.65–1.14	.29	0.90	0.70–1.15	.38
Parity	1.04	0.78–1.38	.81	0.97	0.76–1.25	.83
BHB‐level	0.73	0.48–1.12	.15	0.77	0.53–1.11	.16
Metritis	0.66	0.45–0.95	.03	0.69	0.50–0.96	.03
Subclinical endometritis	1.07	0.73–1.53	.77	1.01	0.74–1.40	.93
Clinical endometritis^¶^	0.76	0.52–1.09	.14	0.74	0.54–1.03	.07
Back fat thickness	0.88	0.59–1.30	.52	0.84	0.60–1.19	.32

† FSCR = First service conception risk.

‡ TCR = Total conception risk

§ Factors: Study group (0: CON; 1: MET), parity (0: 2nd lactation; 1: ≥3rd lactation), BHB‐level at 3, 5 and 8 dpp (0: normoketotic; 1: at least one day hyperketotic, ≥1.2 mmol/L BHB), uterine health status at 5 dpp (0: healthy; 1: metritis), subclinical endometritis (0: no; 1: yes), clinical endometritis (0: no; 1: yes), back fat thickness class at 31 ± 3 dpp (0: ≥14 mm; 1: <14 mm).

* *p* < .05.

### 3.4 Second service conception risk

Days to second service were 96.3 ± 13.3 and 96.4 ± 13.5 for CON and MET, respectively. No significant difference was found in SSCR between CON (43.9%) and MET (46.0%, Table 2). This calculation was based on 123 cows in CON and 113 cows in MET.

### 3.5 Total conception risk

For calculating TCR, 1,185 AI from 916 cows were used (1st AI: 916, 2nd AI: 236, >2nd AI: 33). No significant difference regarding TCR was found between CON (44.4%) and MET (42.1%). In CON, significantly more healthy cows (48.2%) became pregnant compared to cows with CE (35.1%). In MET, cows with SE (47.0%) showed a significantly greater TCR compared to cows with CE (34.5%). Furthermore, TCR in MET was greater for cows with E1 (41.1%) compared to cows with E2 (23.1%). More details are presented in Table 2. The binary logistic regression model revealed metritis also as significant risk factor for TCR (Table 3).

### 3.6 Days open 140

**Figure 1:**
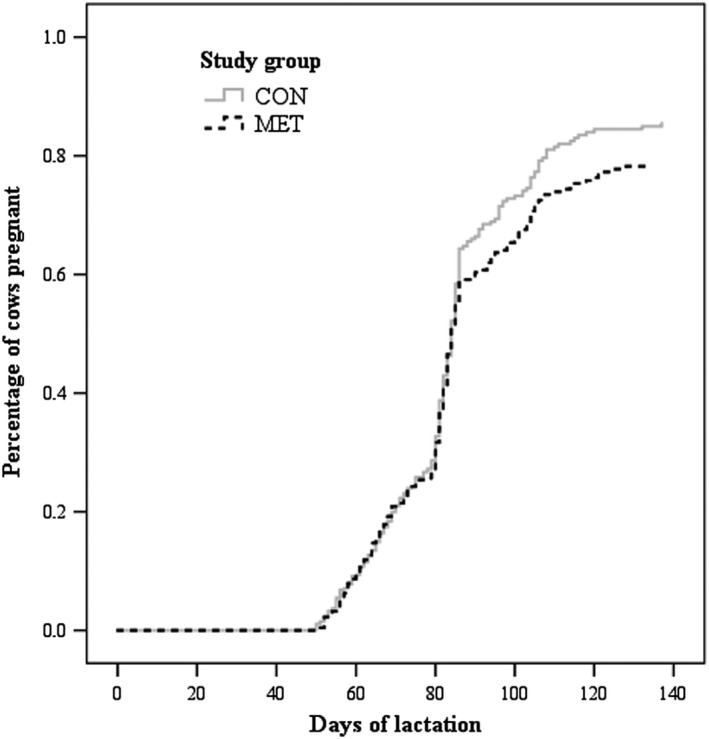
Proportion of pregnant cows for CON (control group) and MET (rumen‐protected methionine group) within 140 days of lactation

DOPN140 was calculated for a total of 513 cows that became pregnant before they left the study pens (CON: *n* = 270; MET: *n* = 243). DOPN140 was 78.1 ± 15.9 and 78.3 ±16.6 in CON and MET, respectively. For the Cox regression analyses (Table 4) and Kaplan–Meier survival analyses (Figure 1), the 916 animals from FSCR were used, censored for cows not pregnant and censored for study animals that were removed from the study after FSCR. Results from Cox regression analyses showed that metritis (HR = 0.70; CI_95_ = 0.54–0.91; *p* = .01) and CE (HR = 0.74; CI_95_ = 0.58–0.95; *p* = .02) were significant factors affecting DOPN140 (Table 4). Figure 1 illustrates the time to pregnancy and proportion of cows pregnant for both study groups.

**Table 4:** Results of cox regression analysis for odds of conception until day 140 of lactation

	Conception†		
Factors‡	Hazard Ratio	95% CI	*P*‐value*
Study group	0.90	0.75–1.08	.25
Parity	0.93	0.77–1.12	.44
BHB‐level	0.77	0.58–1.04	.09
Metritis	0.70	0.54–0.91	.01
Subclinical endometritis	1.10	0.87–1.39	.44
Clinical endometritis	0.74	0.58–0.95	.02
Back fat thickness	0.79	0.61–1.02	.07

† From 916 study animals, 91 dairy cows were not used because of at least one missing result from clinical examination

‡ Factors: Study group (0: CON; 1: MET), parity (0: 2nd lactation; 1: ≥3rd lactation), BHB‐level at 3, 5 and 8 dpp (0: normoketotic; 1: at least one day hyperketotic, ≥1.2 mmol/L BHB), uterine health status at 5 dpp (0: healthy; 1: metritis), subclinical endometritis (0: no; 1: yes), clinical endometritis (0: no; 1: yes), back fat thickness class at 31 ± 3 dpp (0: ≥14 mm; 1: <14 mm).

* *p* < .05.

### 3.7 Pregnancy loss

In total, 22/513 (4.3%) pregnancy losses after the first pregnancy examination were recorded. Of those, nine were diagnosed while the cows were still in the study pens (CON *n* = 4, MET *n* = 5) and 13 after leaving the study group (*n* = 4 in CON, *n* = 9 in MET).

## 4 DISCUSSION

We investigated the effect of supplementing RPM on reproductive performance at the herd level. The effects of supplementing RPM between 21 days before calving to 30 days after calving on preimplantation embryos (Acosta et al., 2016) and on the steroidogenic potential of the first post‐partum dominant follicle (Acosta et al., 2017) have been investigated in the past. Information about the impact of RPM on fertility on a herd level, however, has been published to a lesser extent. A recent paper from Stella et al. (2018) on 20 Holstein cows fed RPM from 21 days prepartum to 73 days post‐partum found no significant difference in the proportion of cows affected by SE at 30 dpp (SE was defined as >18% PMN in a cytological smear), but cows supplemented with RPM tended to have SE less frequently. Because the time between the beginning of supplementation with RPM and the evaluation of uterine health status at 31 ± 3 dpp was too short, we regarded uterine health status not as a result of RPM supplementation but as a potential risk factor for reproductive performance parameters. A significantly lower number of cows affected with SE in CON could be regarded as positive bias for this group. This hypothesis, however, was not tested in this study.

Healthy cows in CON showed better FSSR, FSCR and TCR compared to cows in CON with CE. These findings are in line with previous studies reporting a negative impact of CE on reproductive performance (LeBlanc, 2008; Toni, Vincenti, Ricci, & Schukken, 2015). Interestingly, cows in MET with SE had a better FSSR and TCR compared to cows in MET with CE. Healthy study animals in MET showed only significantly greater FSSR compared to MET cows with CE. FSCR, SSCR or TCR were not significantly different between healthy cows and cows with CE in MET.

Toledo et al. (2017) evaluated pregnancies per AI and found no significant differences between cows supplemented with RPM and animals without supplementation. Similar to that study, FSCR, SSCR and TCR in our study showed no significant differences for CON and MET. Furthermore, DFS and DOPN140 were not affected by supplementation of RPM.

Binary logistic regression analyses showed that metritis affected FSCR and TCR. Cox regression analyses showed that metritis and CE affected the hazard ratio for conception. These findings are in line with other studies that showed that cows affected with endometritis have their reproductive performance substantially impacted (LeBlanc, 2008; Plöntzke, Madoz, De la Sota, Heuwieser, & Drillich, 2011) and metritis, particularly puerperal metritis, correlates with poor reproductive performance (Giuliodori et al., 2013).

In contrast to Toledo et al. (2017), who reported fewer pregnancy losses from 28 to 61 and 32 to 61 days after AI, we found no differences in pregnancy losses between the groups. It has to be noted that the total number of pregnancy losses in our study was low and, thus, statistical power is limited. In our study, the first pregnancy examinations were performed 42 ± 3 days after AI and the second pregnancy check at 93 ± 3 days after AI. Pregnancy losses are confounded by the time between AI and the pregnancy check (Santos, Thatcher, Chebel, Cerri, & Galvão, 2004). Moreover, fewer pregnancy losses are found the later pregnancy is diagnosed. Therefore, it remains unclear if an impact of feeding RPM on pregnancy loss was underestimated in our study.

The hypothesis of our study was that feeding RPM improves reproductive performance at the herd level. This hypothesis, however, was not confirmed by the presented results. One confounding factor was the experimental design of this study, which had the limitation of only one pen with and one pen without supplementation of RPM. This implies that individual cows with low dry matter intake may have lower RPM intake than some cows with greater intake, resulting in a potential wide variation in individual feed intake. Therefore, future studies about the impact of methionine on reproduction could use feeding systems which record the individual feed intake. This was not possible in our study that was designed as field trial with a large number of cows.

A number of meta‐analyses (Patton, 2010; Zanton, Bowman, Vázquez‐Añón, & Rode, 2014) have found positive effects of supplementing methionine, the first‐limiting AA for dairy cattle (NRC, 2001), on milk production. Furthermore, (Peñagaricano et al., 2013) investigated the effect of supplementing methionine on the transcriptome during the periconceptional period and found that many genes that were critical for subsequent embryonic function were decreased by the supplementation of methionine. In contrast, Robinson (2010) investigated the impact of manipulating ration metabolizable AA levels in a systematic review and concluded that the contribution of microbial protein in relation to duodenal protein is quite large. Thus, the extent of restricting one limiting AA is relatively small and even supplementing a specific AA results in only very few benefits. Therefore, the impact of supplementing RPM to compensate potential methionine deficiency on milk production and maybe also the effect of fertility is still controversial.

Furthermore, there is evidence that effects of RPM also depend on the beginning of supplementation. Stella et al. (2018) showed improved uterine immune function when supplementation started at the beginning of the transition period. Additionally, results from Zhou et al. (2016) and Batistel et al. (2017) indicated that peripartal RPM supplementation has positive effects on the performance of dairy cows. Therefore, it can be hypothesized that starting the supplementation of RPM pre‐calving could improve fertility. Another aspect that might contribute to our results is that reproductive performance is highly dependent on farm management. It cannot be excluded that supplementing RPM on a farm with different management strategies and feeding regimes results in improved reproductive performance.

## 5 CONCLUSIONS

Our hypothesis that supplementing RPM has a positive effect on reproductive performance at the herd level was not confirmed by this study. To our knowledge, this is the first study analysing the effects of RPM incorporated into the TMR on reproductive performance with a large number of animals. Uterine diseases had a negative impact on fertility. Future studies should consider supplementing RPM in the transition period, farms at different management levels and with different feeding strategies; thus, presented results should be interpreted with caution.

## CONFLICT OF INTEREST

The authors declared no conflict of interest. Authors A.H. and C.P. are employed by Evonik Nutrition and Care, a company that manufactures and commercializes feed additives, and contributed to the design of the study, feed sample analyses and their interpretation, and preparation of the manuscript. Evonik Nutrition and Care had no influence on data analyses, interpretation and presentation of the results.

## AUTHORS CONTRIBUTIONS

DS was involved in animal sampling, analysed the data and drafted the manuscript. MI, MH, DT, KS, AH, CP and MD designed the study, assisted in data analyses and contributed to drafting the paper. VS, EG, PK, SK, KW, HP and MI assisted in animal sampling and data analyses. MD and MI supervised the study.

## Data Availability

The data that support the findings of this study are available from the corresponding author upon reasonable request.
