# Supplementary material for: Supplementing rumen‐protected methionine to lactating multiparous dairy cows did not improve reproductive performance
Source: Reprod Domest Anim. 2019 Aug 6;54(9):1265–73. doi: 10.1111/rda.13509 (PMC6851860; doi:10.1111/rda.13509)
Supplement: Supplementary file 1 [file RDA-54-1265-s001.docx]

**Supporting information**

**TABLE S1** Ingredient composition for basal diets at the beginning and at end of the study

|  | | Experimental diets | | |
| --- | --- | --- | --- | --- |
| Ingredient, % of DM | | Beginning of the study | | End of the study |
| Corn silage | 35.9 | | 30.6 | |
| Alfalfa silage | 18.2 | | 13.4 | |
| WDGS**^†^** | 9.7 | | - | |
| Corn gluten meal | - | | 8.5 | |
| Corn-Cob mix (CCM) | 9.2 | | 7.0 | |
| Rapeseed extraction meal | 8.3 | | 12.7 | |
| Beet pulp silage | 6.6 | | 10.3 | |
| Sorghum meal | 4.3 | | 6.8 | |
| Bean flakes | - | | 3.4 | |
| Molasses | 3.1 | | 3.1 | |
| Wheat Straw | 1.7 | | 2.4 | |
| Alfalfa haylage | 1.0 | | - | |
| Sodium bicarbonate | 0.4 | | 0.5 | |
| Limestone | 0.4 | | 0.5 | |
| Mineral and vitamin premix^‡§^ | 0.4 | | 0.4 | |
| Soybean meal, 48% CP | 0.4 | | - | |
| Salt | 0.3 | | 0.3 | |
| Urea | 0.1 | | 0.1 | |

Total (%) 100.0 100.0

**^†^**Wet distillers grains with solubles

**^‡^**Contained a minimum per kg of 12.00% Mg, 9.83% S, 1,000 kIU of vitamin A, 200 kIU of vitamin D_3_, 9,000 mg vitamin E, 1,500 mg Cu, 6,500 mg Mn, 30 mg Co, 7,500 mg Zn, 110 mg I, 40 mg Se

**^§^**For the MET study group 1 kg of the mineral and vitamin premix contained an additional amount of rumen-protected methionine of 22.0 ± 3.7%

**TABLE S2** Results of TMR analysis for basal experimental diets in CON and MET (mean ± SD standard deviation)

|  | Experimental diets^†^ | |
| --- | --- | --- |
| Parameter^‡^ | CON^§^ ±SD | MET^¶^ ±SD |
| DM, % of fed | 41.0 ± 1.6 | 41.1 ± 1.3 |
| CP, % of DM | 16.4 ± 0.8 | 16.4 ± 0.6 |
| NDF, % of DM | 34.8 ± 2.8 | 34.8 ± 1.2 |
| ADF, % of DM | 22.1 ± 1.0 | 21.9 ± 0.9 |
| NFC, % of DM | 38.8 ± 3.3 | 38.9 ± 1.1 |
| Fat, % of DM | 3.2 ± 0.2 | 3.1 ± 0.2 |
| Ash, % of DM | 6.7 ± 0.3 | 6.7 ± 0.3 |
| NE_L_, Mcal/kg of DM | 1.7 ± 0.0 | 1.7 ± 0.0 |

^†^Average based on weekly taken samples, composited and analyzed monthly

^‡^DM: dry matter, CP: crude protein, NDF: neutral detergent fiber, ADF: acid detergent fiber, NFC: nonfiber carbohydrates, NE_L_: net energy for lactation

^§^CON = Control group

^¶^MET = Rumen-protected methionine group (supplemented with rumen-protected methionine, 25.0 g to 27.2 g per cow per day)
